# Peripheral Venipuncture in Pediatric Patients: A Mini-Review of Clinical Practice and Technological Advances

**DOI:** 10.3390/jcm14186397

**Published:** 2025-09-10

**Authors:** Luiza Elena Corneanu, Ovidiu Rusalim Petriș, Cătălina Lionte, Mara Sînziana Sîngeap, Eric Oliviu Coșovanu, Sabrina Grigolo, Ivona Andreea Șova

**Affiliations:** 1 Faculty of Medicine, Grigore T. Popa University of Medicine and Pharmacy, 700115 Iași, Romania; luiza-elena.corneanu@umfiasi.ro (L.E.C.); catalina.lionte@umfiasi.ro (C.L.); mara-sinziana.singeap@umfiasi.ro (M.S.S.); cosovanu_eric-oliviu@d.umfiasi.ro (E.O.C.); andreea-ivona_sova@umfiasi.ro (I.A.Ș.); 2 Department of Nursing, School of Medicine, University of Eastern Piedmont “Amedeo Avogadro”, Via del Duomo, 6, 13100 Vercelli, Italy; sabrina.grigolo@aslto3.piemonte.it

**Keywords:** pediatric venipuncture, venous blood collection, children, sample quality, child-centered care, venous ultrasound, near-infrared projection, transillumination

## Abstract

**Background:** Venous blood collection in pediatric patients is a critical procedure for diagnostic and monitoring purposes, yet it remains considerably more challenging than in adults. Factors such as small vein size, limited cooperation, and heightened sensitivity to pain contribute to technical difficulties and increased error rates. **Objectives:** This mini-review aims to provide a concise synthesis of current clinical practices and emerging technologies that support safer, more efficient venipuncture in children. **Results:** Key findings include the anatomical and procedural considerations relevant to pediatric venipuncture, age-specific recommendations for technique and positioning, as well as evidence-based strategies to reduce pain and anxiety. Common preanalytical errors, particularly hemolysis and insufficient sample volumes, are also addressed, along with their implications for clinical outcomes. Recent advances in medical digitalization, including the use of venous ultrasound, near-infrared projection, and transillumination, offer valuable support in overcoming procedural challenges. These technologies are not meant to replace human expertise but to complement it, improving vein visualization and increasing first-attempt success rates when integrated into a child-centered approach. **Conclusions:** Venous blood collection in pediatric patients requires a delicate balance between technical proficiency and human-centered care. Emphasis is placed on the importance of a child-centered approach, combining technical skill with empathy and clear communication. Enhancing the quality and safety of venous sampling in children requires not only training and standardization, but also a deeper understanding of the psychological dimensions involved in pediatric care.

## 1. Introduction

Venous blood sampling is a fundamental diagnostic procedure, offering essential information for clinical decision-making in both acute and chronic pediatric settings. Despite its routine nature in adult populations, venipuncture in children remains a significant clinical and emotional challenge, often associated with procedural pain, anxiety, and increased risk of sampling errors [1].

The pediatric population presents unique anatomical and physiological characteristics that complicate vein access, particularly in infants, toddlers, and uncooperative children. Furthermore, the emotional response to needle-based procedures is amplified in young patients, requiring tailored approaches that minimize discomfort and psychological trauma [2].

In recent years, attention has shifted toward improving the quality and safety of pediatric venipuncture through the development of child-specific techniques, staff training, distraction strategies, and the implementation of evidence-based guidelines. Additionally, alternative sampling methods (e.g., capillary or arterial sampling) are often considered when venous access proves difficult or traumatic [3].

In response to these challenges, recent advances in medical technology have introduced novel tools aimed at improving vein visualization and procedural success in pediatric venous access. Techniques such as venous ultrasound, near-infrared projection, and transillumination enhance the identification of peripheral veins, especially in neonates and infants with limited visible access. These methods are increasingly being integrated into pediatric practice to support more accurate and less traumatic venipuncture, often serving as valuable adjuncts rather than replacements for clinical expertise [4,5,6,7,8].

Furthermore, the development of robot-assisted venous blood collection systems has shown promise in adult care settings, combining imaging technologies with automated cannulation. However, their use in pediatric populations remains limited due to practical barriers such as the need for complete immobility, noise-related distress, and anatomical variability [9,10]. These innovations highlight the growing role of digital health tools in pediatric care, while also emphasizing the continued importance of human adaptability, empathy, and procedural experience.

This mini-review aims to summarize the current knowledge on venous blood collection in children, exclusively on peripheral venipuncture techniques and challenges, excluding central venous access procedures, with a focus on anatomical considerations, recommended techniques, pain reduction strategies, common procedural errors, and recent technological innovations. By presenting both traditional and emerging approaches, the review seeks to provide clinicians with a practical and concise overview to support high-quality, safe, and child-centered venous sampling practices in pediatric populations.

## 2. Discussion

### 2.1. Anatomical and Technical Considerations

Venipuncture in pediatric patients requires careful attention to anatomical variability across age groups. Unlike adults, children, particularly neonates and infants, have smaller and more fragile veins, which are often difficult to palpate and visualize. Common sites for venous access include the dorsal hand veins, antecubital fossa, and, in infants, scalp or foot veins [3,11,12].

The choice of needle gauge is critical; typically, a 23–25 gauge butterfly needle is preferred for small or superficial veins to reduce trauma and hemolysis. Proper positioning and immobilization of the child are equally important to prevent sudden movement and ensure successful collection. The use of transillumination devices or ultrasound guidance may improve vein visualization and first-attempt success rates, especially in patients with difficult access [3,5].

Additionally, skin antisepsis must be performed meticulously using age-appropriate antiseptic agents, such as chlorhexidine or povidone-iodine, with careful consideration of skin sensitivity in neonates. Adequate drying time should be observed to avoid contamination and hemolysis. The volume of blood collected must also be carefully calculated based on the child’s weight and clinical indication, to avoid iatrogenic anemia, particularly in very young patients [3,13].

### 2.2. Age-Specific Techniques and Recommendations

The technique and site of venous blood collection should be tailored according to the child’s age, size, cooperation level, and clinical condition.

In neonates and infants, scalp veins, dorsal hand veins, and the great saphenous vein are frequently used. Scalp vein access, although more invasive in appearance, offers stability and can be easier to secure in neonates. Heel pricks are often preferred for capillary sampling in this age group, but when venous access is necessary, utmost care must be taken to minimize trauma [3,4].

For toddlers and preschool-aged children, peripheral veins in the upper extremities, such as the antecubital or cephalic vein, are commonly used. However, due to increased mobility and fear, additional support from caregivers or trained personnel for gentle restraint may be required. Distraction techniques (e.g., animated videos, toys, or music) can improve cooperation and reduce distress [3,14].

In school-aged children and adolescents, standard adult venipuncture techniques are often applicable, provided clear communication and reassurance are offered. Allowing the child some control over the situation, such as choosing the arm or position, can empower them and reduce anxiety [3,14].

Regardless of age, the use of topical anesthetics or cold sprays is encouraged to minimize pain, especially when time allows. Evidence-based protocols that match technique with developmental stage significantly improve success rates and overall experience for the child and caregivers [15].

To facilitate clinical decision-making regarding site selection, Table 1 provides a simplified overview of preferred peripheral venous access sites in pediatric patients, categorized by age group and approximate weight. This stratification is based on anatomical feasibility and commonly reported clinical practice.

### 2.3. Pain and Anxiety Management

Pain and anxiety during venous blood collection represent major barriers to procedural success and can lead to long-term fear of medical settings in pediatric patients. Compared to adults, children have a lower pain threshold and a limited ability to rationalize the necessity of blood sampling, which amplifies the emotional response [14,15].

Several non-pharmacological strategies have proven effective in reducing distress. Distraction techniques, such as interactive screens, cartoons, or storytelling, can redirect attention away from the procedure. For infants, oral sucrose or breastfeeding during the procedure has demonstrated analgesic effects. Older children may benefit from guided breathing exercises or choosing a preferred coping strategy [15].

Parental presence plays a crucial role; calm and supportive caregivers can positively influence the child’s emotional state. However, it is important to prepare both the child and the parent in advance, as anxious caregivers may inadvertently heighten the child’s distress [15].

Topical anesthetics, such as lidocaine–prilocaine creams, are safe and effective in reducing procedural pain, particularly when applied 30–60 min before venipuncture. In settings where time is limited, vapocoolant sprays may offer rapid analgesia [15].

Staff training is essential to ensure a child-friendly approach, including the use of simple, reassuring language and the avoidance of threatening terminology. A calm, confident, and empathetic attitude by the healthcare provider fosters trust and increases the likelihood of a successful first attempt [15].

### 2.4. Procedural Errors

Venous blood collection in pediatric patients carries an inherently higher risk of procedural errors compared to adults, primarily due to anatomical constraints, limited patient cooperation, and smaller blood volumes. Among the most frequent issues are hemolysis, insufficient sample volume, clotted specimens, and failed venipuncture attempts [2,3].

Hemolysis is a common preanalytical error in pediatric blood samples and may result from excessive suction force during collection, inappropriate needle gauge, prolonged tourniquet time, or vigorous mixing of blood with additives. Hemolyzed samples can alter laboratory parameters, thereby compromising result accuracy and necessitating repeated procedures [2].

Sampling failures, including multiple puncture attempts or inability to access the vein, may delay diagnosis and erode patient and caregiver trust. These failures are more likely in children with dehydration, obesity, chronic illness, or difficult vein anatomy. In such cases, escalation to more experienced clinical staff, use of vein-finding devices, or considering alternative access routes (e.g., capillary sampling, central line draws if available) may be warranted [3,12].

Insufficient sample volume, a common problem in infants and neonates, may prevent completion of all requested analyses, resulting in additional blood draws. It is essential to calculate blood volume limits based on body weight (usually not exceeding 3–5% of total blood volume over 24 h), especially in critically ill or underweight patients [3,13].

Training programs for pediatric venipuncture, including simulation-based modules, have been shown to reduce error rates and increase first-attempt success. Institutions should implement standardized protocols and regular audits of sample rejection rates to ensure quality assurance in pediatric blood collection [2].

### 2.5. Technological Innovations and the Future of Pediatric Venous Access

Given the anatomical and behavioral challenges specific to pediatric patients, especially neonates and infants, the use of adjunct technologies has been explored to improve venous access success.

Venous ultrasound has proven effective in identifying deeper veins, particularly in neonates with poor peripheral access. Although not routinely used for standard venipuncture, ultrasound guidance is increasingly employed in neonatal intensive care units for difficult cases or central venous access [4,5,6].

Near-infrared projection devices and transillumination have emerged as valuable tools in locating superficial veins by enhancing visualization through tissue. These technologies are particularly helpful in infants and toddlers, where veins may be difficult to palpate or to visualize without assistive devices. Near-infrared-based devices project an image of the subcutaneous vasculature onto the skin, aiding clinicians in choosing the optimal puncture site and reducing the number of failed attempts [7,8].

In this context, the concept of “one-prick” venipuncture, achieving successful blood collection on the first attempt, has emerged as a critical quality benchmark in pediatric care. Repeated puncture attempts not only cause physical discomfort and procedural anxiety, but they are also associated with increased rates of hemolysis, insufficient samples, and diagnostic delays.

Studies suggest that first-attempt success rates vary widely, ranging from 60% to 80% in general pediatric populations, and dropping to as low as 30–40% in patients classified as having difficult intravenous access. Factors such as young age, poor vein visibility, dehydration, or obesity contribute significantly to this variability [16].

Implementing supportive technologies, such as ultrasound, near-infrared imaging, and transillumination, has been shown to improve these outcomes, particularly when combined with proper training and child-friendly procedural techniques. Striving for a “one-prick” approach not only enhances procedural success but also reflects a broader commitment to minimizing trauma and promoting patient-centered care in pediatric practice [17,18].

While no universal benchmarks are established in guidelines, published data indicate that first-attempt success rates in pediatric populations depend on clinical setting and provider expertise. Adjunctive technologies such as ultrasound guidance have shown to improve first-attempt success rates by 15–30%, particularly in patients with difficult intravenous access. Near-infrared projection and transillumination offer additional support in visualizing superficial veins, with more modest improvements ranging from 5% to 20%, especially in infants and toddlers [16,18]. These findings support the integration of such tools into evidence-based protocols aimed at improving procedural outcomes in pediatric venipuncture.

In parallel, the development of venipuncture robots has advanced considerably in adult care, with systems capable of vein detection, cannula insertion, and blood collection. However, their application in pediatrics remains extremely limited due to several factors: the need for the child to remain completely still, the distress caused by the device’s noise or mechanical components, and the variability of vein size and position in young patients [9,10]. These limitations highlight the importance of human judgment, tactile feedback, and child-centered communication in pediatric procedures, elements not easily replaced by automation. At present, no large-scale clinical trials have demonstrated safety or efficacy in pediatric populations, and these technologies are not part of routine laboratory practice.

To better illustrate the clinical utility and limitations of these technologies, Table 2 summarizes their main advantages and pediatric-specific applications [19]. By offering a comparative perspective, this overview highlights how such adjuncts can complement clinical judgment and procedural expertise, particularly in vulnerable populations such as neonates and young children.

Considering the essential role of venous access in pediatric care, future efforts must focus on refining existing technologies and forming dedicated vascular access teams or associations, as seen in adult critical care settings (e.g., Association for Vascular Access—AVA [20]; it has a pediatric section—Pediatric and Neonatal Special Interest Group [21]). Such interdisciplinary groups, comprising pediatric nurses, physicians, and vascular access specialists, could improve first-attempt success rates, standardize training, and ensure safer, less traumatic experiences for children.

Looking ahead, non-invasive smart technologies, such as wearable sensors, sweat-based biosensors, and optical diagnostic devices, have emerged as attractive alternatives to traditional blood sampling. Their appeal lies in improved patient comfort and potential for continuous, real-time monitoring. However, current evidence suggests that these systems remain limited in both diagnostic scope and measurement accuracy, especially in pediatric populations [22]. Devices designed to estimate blood biomarkers like hemoglobin or glucose often lack the reliability required for clinical decision-making [23,24]. As such, they function as adjunct tools rather than viable replacements for venous blood collection.

Recent studies have increasingly evaluated the clinical effectiveness of adjunct technologies in improving pediatric venous access, with variable results depending on patient age and clinical setting. Ultrasound-guided venipuncture demonstrates the strongest evidence, with several randomized trials and meta-analyses showing significantly higher first-attempt success rates, particularly in neonates and children with difficult intravenous access [25,26,27]. Conversely, near-infrared projection devices and transillumination techniques provide valuable support in visualizing superficial veins, but their overall impact on procedural success remains inconsistent across studies [28,29]. Table 3 summarizes the available evidence from randomized controlled trials, meta-analyses, and systematic reviews, highlighting comparative success rates, study sizes, and clinical applicability for each technique. This evidence-based synthesis emphasizes the importance of integrating such technologies thoughtfully, combining technological advancements with clinical expertise to optimize patient outcomes.

Continued innovation in this space is essential, but its integration into pediatric care must be guided by rigorous validation studies and consideration of age-specific challenges. For the foreseeable future, venipuncture remains indispensable, particularly when comprehensive laboratory analysis is required.

## 3. Conclusions

Venous blood collection in the pediatric population remains a complex yet essential component of modern diagnostic medicine. While the procedure is routinely performed, it poses distinct challenges in children due to anatomical, physiological, and emotional factors. A successful pediatric venipuncture requires not only technical proficiency, but also a patient-centered approach that minimizes pain, anxiety, and procedural errors.

Adapting techniques to the child’s age and condition, using appropriate tools and analgesic strategies, and ensuring effective communication with both children and caregivers are key to improving the quality and safety of the procedure. Moreover, reducing preanalytical errors through staff training, protocol adherence, and careful handling of samples contributes significantly to diagnostic accuracy and overall patient satisfaction.

As efforts continue to make healthcare more child-friendly, the refinement of venous blood collection practices must remain a priority. Future research should focus on standardizing pediatric-specific protocols, integrating emerging technologies (such as portable vein finders or automated collection systems), and exploring the psychological dimensions of procedural care.

From a practical standpoint, clinicians may benefit from a structured, stepwise approach to pediatric venous blood collection. Initial attempts should rely on traditional techniques with age-appropriate positioning and distraction methods. If access is not achieved within two attempts or in patients with known difficult intravenous access, escalation to adjunct technologies such as transillumination or near-infrared projection is recommended. In neonates or critically ill children with poor peripheral veins, early use of ultrasound guidance should be considered. Staff training and clear escalation pathways can help reduce procedural delays, improve first-attempt success, and avoid unnecessary trauma. Developing and adopting institution-level algorithms for pediatric venipuncture may further enhance standardization and outcomes.

Considering its critical role in pediatric diagnostics, venous blood collection warrants the implementation of tailored strategies, ranging from trained medical staff and adjunctive technologies to the creation of specialized pediatric vascular access teams. Balancing technological innovation with empathetic, child-centered care remains essential for enhancing procedural success and ensuring a positive experience for young patients.

## Figures and Tables

**Table 1:** Selection of peripheral venous access sites in pediatric patients.

Age Group	Weight Range	Preferred Venous Sites
Neonates (0–28 days)	<4 kg	Scalp veins, dorsal hand veins, great saphenous vein
Infants (1–12 months)	4–10 kg	Dorsal hand veins, antecubital fossa, cephalic veins
Toddlers (1–3 years)	10–20 kg	Dorsal hand veins, cephalic and basilic veins
Preschool (3–6 years)	15–25 kg	Dorsal hand veins, antecubital fossa (cephalic, basilic)
School-aged (6–12 years)	20–40 kg	Antecubital veins, dorsal hand veins
Adolescents (>12 years)	>40 kg	Forearm veins, antecubital fossa (adult protocol)

**Table 2:** Advantages of adjunct technologies in pediatric venous blood collection.

Technology	Primary Advantages	Pediatric-Specific Benefits	Limitations
Venous Ultrasound	Real-time visualization of deep and superficial veins	Increases success in neonates and children with difficult access	Requires training; less accessible in non-hospital settings
Near-Infrared Projection	Projects vein map on skin using hemoglobin absorption contrast	Non-invasive, improves first-attempt success, child-friendly	Limited depth penetration; less effective on dark or edematous skin
Transillumination	Illuminates tissues to enhance visibility of superficial veins	Especially useful in infants and small children with thin skin	Lower resolution; works best in extremities (hands/feet)
Robot-Assisted Collection	Automated detection, cannulation, and blood draw with imaging guidance	Potential to reduce operator dependency and standardize procedures	Impractical in children due to movement, distress, and size variability

**Table 3:** Evidence summary for adjunct technologies in pediatric venous blood collection.

Technology	Study Type	Sample Size	First-Attempt Success (Intervention vs. Control)	Key Findings
Ultrasound	Randomized cohort	110	90% vs. 18%	Significantly improved success rates with ultrasound guidance
Ultrasound	Meta-analysis	—	RR = 1.53 (95% CI: 1.14–2.04)	Pooled data favors ultrasound-guided techniques
Ultrasound in Neonates	Systematic review	11	>85% (ultrasound) vs. lower with standard technique	Consistently high success in neonates using ultrasound
Near-Infrared Projection	Randomized controlled trial	123	72% (near-infrared) vs. 79% (standard)	No significant improvement in overall success, useful in selected cases
Near-Infrared Projection	Systematic review	—	No significant effect	It does not significantly reduce first-attempt failure rates

## Data Availability

Not applicable.

## References

[1] Şenol Ş. (2025). Preanalytical errors in pediatric blood sampling: A systematic review of common challenges and risks. Turk. J. Biochem..

[2] Riddick L. (2023). Paediatric blood sampling: How to improve your chances of getting it right. Paediatr. Child Health.

[3] Hjelmgren H., Ygge B.M., Nordlund B., Andersson N. (2022). Nurses’ experiences of blood sample collection from children: A qualitative study from Swedish paediatric hospital care. BMC Nurs..

[4] Pittiruti M. (2012). Ultrasound Guided Central Vascular Access in Neonates, Infants and Children. Curr. Drug Targets.

[5] Brusciano V., Lecce M. (2023). Advantages of the use of ultrasound in newborn vascular access: A systematic review. J. Ultrasound.

[6] Schiefer J., Lichtenegger P., Zimpfer D., Hutschala D., Kuessel L. (2021). Performing central venous catheters in neonates and small infants undergoing cardiac surgery using a wireless transducer for ultrasound guidance: A prospective, observational pilot study. BMC Pediatr..

[7] Fehr G., Rigali M., Weller G., Grap S.M., Coleman M. (2023). Efficacy of Infrared Vein Visualization versus Standard Technique for Peripheral Venous Cannulation in Infant and Toddler Populations: A Randomized Study. Children.

[8] Ng S.L.A., Leow X.R.G., Ang W.W., Lau Y. (2024). Effectiveness of near-infrared light devices for peripheral intravenous cannulation in children and adolescents: A meta-analysis of randomized controlled trials. J. Pediatr. Nurs..

[9] Wang Y., Ruan H., Ruan Y., Li Q., Wang C. (2025). Intelligent blood collection robot: Key technologies, clinical applications, and future challenges. Front. Bioeng. Biotechnol..

[10] Xue H., Han H., Man G., Feng Z., Yu P. (2025). Design and Analysis of a Portable Robot for Venous Blood Sampling. Machines.

[11] Malinowski M. (2020). Capillary Blood Sampling Procedure in Pediatric Population. Am. J. Biomed. Sci. Res..

[12] Elsevier Elsevier–Clinical Skills|Blood Specimen Collection: Venipuncture (Pediatric). https://elsevier.health/en-US/preview/blood-specimen-collection-venipuncture-pediatric.

[13] Broder-Fingert S., Crowley W.F., Boepple P.A. (2009). Safety of frequent venous blood sampling in a pediatric research population. J. Pediatr..

[14] Revell J., Backman C., Vasily S., Harrison D. (2021). A Cross-Sectional Review of Pain Management Interventions Used for Painful Pediatric Blood Draw Procedures in Hospital. Pain Manag. Nurs..

[15] World Health Organization (2010). WHO Guidelines on Drawing Blood: Best Practices in Phlebotomy.

[16] Schults J.A., Kleidon T.M., Gibson V., Ware R.S., Monteagle E. (2022). Improving peripheral venous cannula insertion in children: A mixed methods study to develop the DIVA key. BMC Health Serv. Res..

[17] Pokala P., Tamilarasan P. (2025). Near infra-red device for difficult intravenous cannulations in children: A boon?. Sri Lanka J. Child Health.

[18] Kim M.J., Park J.M., Rhee N., Je S.M., Hong S.H. (2012). Efficacy of VeinViewer in pediatric peripheral intravenous access: A randomized controlled trial. Eur. J. Pediatr..

[19] YouTube Video: Association for Vascular Access. IV League-3rd Session. Vein Visualization, Ultrasound, Near Infrared (NIR). Published 31 May 2023. https://www.youtube.com/watch?v=Y38K9jFvi7s.

[20] Association for Vascular Access. https://www.avainfo.org/.

[21] (2025). Pediatric Special Interest Group-Association for Vascular Access. https://www.avainfo.org/page/pedineosig.

[22] Prakashan D., P R R., Gandhi S. (2023). A Systematic Review on the Advanced Techniques of Wearable Point-of-Care Devices and Their Futuristic Applications. Diagnostics.

[23] Min S., Geng H., He Y., Xu T., Zhang X. (2025). Minimally and non-invasive glucose monitoring: The road toward commercialization. Sens. Diagn..

[24] Zhang W., Xiong K., Zhu C., Evans R., Zhou L. (2024). Promoting child and adolescent health through wearable technology: A systematic review. Digit. Health.

[25] D’Alessandro M., Ricci M., Bellini T., Chianucci B., Calevo M.G., Piccotti E., Moscatelli A. (2023). Difficult Intravascular Access in Pediatric Emergency Department: The Ultrasound-Assisted Strategy (DIAPEDUS Study). J. Intensive Care Med..

[26] Ye X., Li M. (2022). Comparison of Ultrasound Guided and Conventional Techniques for Peripheral Venous Catheter Insertion in Pediatric Patients: A Systematic Review and Meta-Analysis of Randomized Controlled Trials. Front. Pediatr..

[27] Casal-Guisande C., López-Domene E., Fernández-Antorrena S., Fernández-García A., Torres-Durán M., Casal-Guisande M., Fernández-Villar A. (2025). Peripheral Vascular Access in Infants: Is Ultrasound-Guided Cannulation More Effective than the Conventional Approach? A Systematic Review. Medicina.

[28] Perry A.M., Caviness A.C., Hsu D.C. (2011). Efficacy of a Near-Infrared Light Device in Pediatric Intravenous Cannulation. Pediatr. Emerg. Care.

[29] Anazi A., Woodman A., Zahrani A., Alsanad M.A., Alzahrani M.S., Alanazi F.R., Rasheed M. (2023). Literature review on the efficacy of near-infrared device in improving peripheral venous access time and number of attempts in pediatric patients. Curr. Med. Res. Opin..

